# Photobiomodulation therapy promotes the ATP‐binding cassette transporter A1‐dependent cholesterol efflux in macrophage to ameliorate atherosclerosis

**DOI:** 10.1111/jcmm.16531

**Published:** 2021-05-05

**Authors:** Qianxia Yin, Haocai Chang, Qi Shen, Da Xing

**Affiliations:** ^1^ MOE Key Laboratory of Laser Life Science & Institute of Laser Life Science College of Biophotonics South China Normal University Guangzhou China; ^2^ Guangdong Provincial Key Laboratory of Laser Life Science College of Biophotonics South China Normal University Guangzhou China

**Keywords:** atherosclerosis, ATP‐binding cassette transporter A1, cholesterol efflux, foam cells, photobiomodulation therapy, reverse cholesterol transport

## Abstract

Atherosclerosis is a chronic inflammatory disease related to a massive accumulation of cholesterol in the artery wall. Photobiomodulation therapy (PBMT) has been reported to possess cardioprotective effects but has no consensus on the underlying mechanisms. Here, we aimed to investigate whether PBMT could ameliorate atherosclerosis and explore the potential molecular mechanisms. The *Apolipoprotein E* (*ApoE*)^−/−^ mice were fed with western diet (WD) for 18 weeks and treated with PBMT once a day in the last 10 weeks. Quantification based on Oil red O‐stained aortas showed that the average plaque area decreased 8.306 ± 2.012% after PBMT (*P* < .05). Meanwhile, we observed that high‐density lipoprotein cholesterol level in WD + PBMT mice increased from 0.309 ± 0.037 to 0.472 ± 0.038 nmol/L (*P* < .05) compared with WD mice. The further results suggested that PBMT could promote cholesterol efflux from lipid‐loaded primary peritoneal macrophages and inhibit foam cells formation via up‐regulating the ATP‐binding cassette transporters A1 expression. A contributing mechanism involved in activating the phosphatidylinositol 3‐kinases/protein kinase C zeta/specificity protein 1 signalling cascade. Our study outlines that PBMT has a protective role on atherosclerosis by promoting macrophages cholesterol efflux and provides a new strategy for treating atherosclerosis.

## 1 INTRODUCTION

Atherosclerosis is the mainly underlying pathology that causes cardiovascular diseases (CVD),^1^ and it results from a maladaptive inflammatory response that is initiated by the intramural retention of cholesterol‐rich, ApoB‐containing lipoproteins in susceptible areas of the arterial vasculature.^2^ The stranded lipoproteins are susceptible to various modifications, which trigger the activation of the vascular endothelial cells and drive monocytes influx into the vascular intima. Monocytes differentiate into macrophages and phagocytize modified lipoproteins by scavenger receptors (SRs) and then shift to a foam cell phenotype.^3^

High‐density lipoprotein cholesterol (HDL‐C) levels are inversely correlated with the CVD risk resulting from atherosclerosis. A major function of HDL is to promote cholesterol efflux and reverse cholesterol transport (RCT).^4^ It is well‐established that foam cells formation and further accumulation in the subendothelial space of the vascular wall are the hallmark of atherosclerotic plaques.^5^ Multiple lines of evidences support that enhancing foam cells cholesterol efflux by HDL particles, the first step of RCT, is a promising anti‐atherogenic strategy.^6^ The importance of ATP‐binding cassette transporter A1 (ABCA1) and ATP‐binding cassette transporter G1 (ABCG1) in mediating lipid efflux from macrophages to HDL has been showed in many studies. Especially, ABCA1 plays a major role in cholesterol homeostasis and HDL metabolism.^7^ Mutations in ABCA1 caused Tangier disease and exhibited a reduced cholesterol efflux, low HDL‐C level and an increased risk to develop atherosclerosis.^8^, ^9^ Moreover, overexpression of ABCA1 increased cholesterol efflux from macrophages and protected against atherosclerosis.^10^

Photobiomodulation therapy (PBMT) using a low‐energy density visible to near‐infrared laser is an innovative physiotherapy. An increasing number of experimental and clinical studies have demonstrated that PBMT played a positive impact in reducing pain and inflammation, promoting wound healing, tissue regeneration and angiogenesis.^11^, ^12^, ^13^, ^14^, ^15^, ^16^, ^17^ It has been reported that PBMT activated cytochrome c oxidase in the mitochondrial respiratory chain and improved mitochondrial function.^18^, ^19^ Researchers also found that PBMT bolstered mitochondrial biogenesis and stimulated ATP synthesis to enhance platelet production.^20^ Besides regulating mitochondrial function, PBMT could activate signalling transduction pathways to modulate the protein expression. For instance, PBMT was reported to rescue dendrite atrophy via up‐regulating BDNF expression.^21^ Some research evidence indicated that PBMT had a certain protective effect on cardiovascular disease. For example, other researchers and our previous works have reported that PBMT enhanced production of vascular endothelial growth factor and promoted angiogenesis.^22^, ^23^ Liu et al^24^ have found that intranasal PBMT improved blood lipids and haemorheology in patients with coronary heart disease or cerebral infarction; Lilach Gavish's group also showed that PBMT enhanced the blocking of abdominal aortic aneurysms by collagen matrix reinforcement in *Apolipoprotein E* (*ApoE*)^−/−^ mice.^25^ The above results suggest that PBMT has the potential to treat atherosclerosis.

The goal of this study is to investigate whether PBMT can protect against atherosclerosis and explore its molecular mechanisms. Here, we found that PBMT could relieve western diet (WD)‐induced atherosclerosis. Treating *ApoE* ^−/−^ mice with PBMT could reduce the atherosclerotic lesion area, normalize the plasma lipid levels including lowering total cholesterol level and LDL‐C level and increasing HDL‐C level. Further results showed that PBMT could promote cholesterol efflux from macrophages through up‐regulating the ABCA1 expression. We demonstrated that the activation of phosphatidylinositol 3‐kinases/protein kinase C zeta/specificity protein 1 (PI3K/PKCζ/SP1) signalling cascade by PBMT was crucial in up‐regulating ABCA1 expression. The study may offer a viable method to control the progress of atherosclerosis.

## 2 MATERIALS AND METHODS

### 2.1 Chemicals

The following reagents were used: wortmannin (WMN, 200 nmol/L, MCE); GÖ6983 (4 µmol/L, MCE); API‐2 (1 µmol/L, MCE); mithramycin A (MTA, 200 nmol/L, Enzo); T0901317 (3 µmol/L, Sigma); 4,4′‐diisothiocyanatostilbene‐2,2′‐disulfonic acid disodium salt (DIDS, 400 µmol/L, MCE); oxidized low‐density lipoprotein (oxLDL; 50 µg/mL, Yiyuan Biotechnologies); HDL (30 µg/mL, Yiyuan Biotechnologies) and apolipoprotein A‐I (ApoA‐I; 15 µg/mL, Sigma).

The following antibodies were used: anti‐ABCA1 (ab18180, abcam); anti‐ABCG1 (ab52617, abcam); anti‐β‐actin (sc‐47778, santa cruz); anti‐SP1 (NB600‐232, Novus Biologicals); anti‐PKCζ (26899‐1‐AP, proteintech; sc‐17781, santa cruz); anti‐pPKCζ (9378, CST); anti‐α‐SMA (14395‐1‐AP, proteintech); anti‐CD11b (ab184308, abcam); anti‐GADPH (sc‐47724, santa cruz); anti‐SR‐BI (21277‐1‐AP, proteintech); anti‐CD36 (18836‐1‐AP, proteintech) and anti‐SR‐AI (sc‐166184, santa cruz).

### 2.2 Animals

The present study was performed in accordance with the NIH Guide for the Care and Use of Laboratory Animals. It was approved by the Institutional Animal Care and Use Committee of our university (South China Normal University, Guangzhou, China). C57BL/6J and *ApoE* ^−/−^ (T001458) mice were purchased from Nanjing Biomedical Research Institute of Nanjing University (NBRI) and kept under stable temperature and humidity in a 12 hours dark/light cycle. The water and cages were sterilized, and the cages were replaced weekly. For consistency, we used male mice in all experiments.

The *ApoE* ^−/−^ mice induced by a high‐fat diet in mice are widely recognized and used for atherosclerotic disease research.^26^, ^27^

*ApoE* ^−/−^ atherosclerosis mouse model: 8‐week‐old *ApoE* ^−/−^ mice were fed with a WD containing 15% fat, and 0.5% cholesterol for 18 weeks. We started PBMT or control treatment after 8 weeks. Body weights and fasting glucose were measured once in 2 weeks. Food and water intake were measured daily. The mice were killed (butorphanol, 2.5 mg/kg, i.p.) after the indicated treatments completed; tissue samples and blood were collected immediately.

### 2.3 PBMT treatment in vitro and in vivo

All cells cultured 6 or 12 hours for RNA or protein analysis after treated with various chemicals and/or irradiated with a 635 nm CW semiconductor laser (NL‐FBA‐2.0‐635, nLight Photonics Corporation, Vancouver, WA) at a power density of 5‐20 mW/cm^2^, 5 minutes. The chemicals were added to the culture medium 30 minutes before PBMT. For protein phosphorylation detection, cells were treated with PBMT again 30 minutes before adding lysate. The entire process was implemented at room temperature. In each experiment, the cells were kept in an absolute dark or a very dim environment, except for subjecting to the laser irradiation, to minimize the ambient light interference.

For in vivo experiments, the abdominal and thoracic hairs of the mice were removed, and then irradiated with PBMT on abdomen and thorax for 10 minutes once a day. We measured the rate of laser intensity loss through the abdominal wall of the mouse before irradiation, approximately 5.823 times. Total laser fluence delivered to the abdominal surface was 58.23 mW/cm^2^, equivalent to a dose of 10 mW/cm^2^ reaching the peritoneal cavity. The control group remained in the specially designed device for the same time as the irradiated group, but the laser source was not activated.

### 2.4 Lipids and lipoprotein measurements

Mice were fasting for 12‐14 hours before the blood samples were collected from heart. Then, centrifuge plasma and store in −80°C. Plasma total cholesterol, triglyceride, LDL‐C and HDL‐C level were measured according to the manufacture instructions in enzymatic measurement. Cholesterol (MAK043) and triglyceride (MAK266) quantitation kit were purchase from Sigma‐Aldrich. LDL‐C and HDL‐C quantitation kit were purchase from Nanjing Jiancheng Bioengineering Institute.

### 2.5 Histology and morphometric analyses

Perfused the mouse hearts with 10 mL of PBS and then left in 20 mL of 4% paraformaldehyde (PFA) at 4°C overnight. Next, hearts were put in 15%‐30% gradient sucrose solution until the tissue sink. Finally, hearts were embedded in OCT, snap‐frozen and stored at −80°C. Slice a series of slices at a thickness of 8 µm with a Leica cryostat, starting at the aortic arch and progressing through the aortic valve area into the heart, and placed on slides.

Unbiased stereological techniques are the gold standard for routine examination of morphological tissue changes in the regulatory or academic environment. Stereology is design based rather than assumption based and uses stringent sampling methods to obtain accurate and precise 3‐dimensional information using geometrical and statistical principles.^28^, ^29^ But, there is no asana synthesis software in our laboratory, we cannot convert multiple plane counts into 3‐dimensional counts. So, we used the average value of statistical serial sections. Every fourth slide from the same mouse serial sections was stained with Haematoxylin and eosin (H&E) and Oil red O (ORO, O1391; sigma) for quantification of lesion area. This method is also widely used in the experiment of statistical plaque size.

Cross sections were stained with antibodies and reagents specific for ABCA1, α‐SMA, CD11b or DAPI and processed for immunofluorescence using standard procedures. For *en face* analysis, the entire aorta of each group of mice was opened longitudinally and stained with ORO. Digitized the photographs of the stained specimens for data analysis and quantified lumen lesion surface area by using Image J software. Data were shown as the percentage of the aorta with positive ORO staining.

### 2.6 Cell culture

Peritoneal macrophages from 6 to 8‐week‐old male C57BL/6J mice were gathered by peritoneal lavage 3 days after intraperitoneal injection of starch broth solution (6% w/v). Cells were cultured in RPMI 1640 supplemented with 10% FBS, penicillin/streptomycin mix (50 mg/mL) in an incubator containing 5% CO_2_ at 37°C. Washed out the non‐adherent cells after 4 hours, and incubated macrophages in fresh RPMI 1640 medium.

### 2.7 Foam cell formation assay

Peritoneal macrophages were cultured and then treated with oxLDL (50 µg/mL) for 24 hours. Next, the cells were washed three times with PBS and cultured in fresh medium for 2 hours, and then treated with PBMT. After 24 hours, discarded the medium and washed with PBS, fixed with 4% PFA for 15 minutes and then stained with BODIPY (Boron‐dipyrromethene, 493/503, 10 μg/mL; Invitrogen). The intracellular lipid droplets were detected with laser scanning confocal microscopy (ZEISS, LSM880).

### 2.8 Cholesterol efflux assays

Peritoneal macrophages were seeded in 12‐well plates (0.5 × 10^6^ cells/well) and cultured overnight. After pretreatment with 22‐NBD Cholesterol (5 µmol/L; N‐1148, Life Technologies) for 24 hours, wash the cells twice with PBS and incubate with fresh medium for 2 hours. Peritoneal macrophages were treated with 635 nm laser irradiation later after replacing with a fresh medium and then ApoA‐I (15 µg/mL) or HDL (30 µg/mL) was added to the fresh medium or not. After incubation for an additional 6 hours, transferred the mediums in each group to a fresh 96‐well plate and measured the fluorescence intensity using a microplate reader (Fm, *λ*_ex_ = 453 nm, *λ*_em_ = 546 nm). The cells in each group were lysed in a lysis buffer, and the lysate was added to another 96‐well plate. It also set the groups treated without adding lipid acceptor, but with PBMT as blank control. Measured the fluorescence after mixing the contents of the wells, (Fc, *λ*_ex_ = 453 nm, *λ*_em_ = 546 nm). The per cent cholesterol efflux was calculated as follows: cholesterol efflux =100% × Fm/(Fc + Fm) ‐ 100% × Fm/(Fc + Fm)_blank_.

### 2.9 RNA isolation and quantitative real‐time PCR

Total RNA was isolated from mouse primary macrophages and aortas using RNAiso Plus (TaKaRa, D9108A) according to the manufacturer's protocol. RNA quantity and purity were assessed using Nanodrop 1000 spectrophotometer (Thermo Scientific), confirming 260/280 ratio of 1.8 to 2.1 for all samples. 1 μg of total RNA was used for reverse transcription using the ReverTra Ace^®^ qPCR RT Master Mix with gDNA Remover (TOYOBO, FSQ‐301). The primers (Table S1) were used. Quantitative real‐time PCR was performed using Applied Biosystems^™^ SYBR^™^ Green (Thermo Scientific^™^) on a Real‐Time Detection System (BioRad). The mRNA level was normalized to β‐actin.

### 2.10 Western blotting

For the Western blot (WB) assay, tissues or cells were lysed with ice‐cold buffer containing 125 mmol/L NaCl, 50 mmol/L Tris‐HCl (pH 7.5), 1% NP‐40, 1.5 mmol/L NaP, 5.3 mmol/L NaF, 100 μg/mL PMSF and 1 mmol/L orthovanadate and supplemented with protease inhibitor cocktail for 60 minutes on ice. The solubilized proteins were quantified by the Bradford assay after centrifugation (4°C, 13 200 g, 20 minutes), separated on an 8% or 10% SDS‐PAGE gel and transferred to a PVDF membrane (Millipore). Blocking the membranes used a TBST solution (150 mmol/L NaCl, 10 mmol/L Tris‐HC [pH 7.4], 0.1% Tween 20) containing 5% non‐fat milk for 1 hour, and then incubated with a specific primary antibody and secondary antibody. An Odyssey infrared imaging system (LI‐COR Biosciences) was used to detect the signals.

### 2.11 Immunofluorescence

For proteins localization or expression experiments, peritoneal macrophages or sections were fixed with 4% PFA on ice for 15 minutes, permeated with TBST (150 mmol/L NaCl, 10 mmol/L Tris‐HCl, pH 7.4, 0.2% Triton X‐100) for 30 minutes, blocked with 5% BSA for 1 hour and stained with designated primary antibody and secondary antibody. All images were analysed using a confocal microscope. Image J was used to analyse original images.

### 2.12 Immunoprecipitation

Immunoprecipitated the cell lysates with anti‐SP1 antibody followed by protein A+G‐Sepharose beads (Roche) at 4°C overnight. The beads were washed with lysis buffer. Resuspend the pellet in the same volume of SDS sample buffer and boiled to remove the Sepharose beads. The whole cell lysates and immunoprecipitated proteins were then analysed by WB.

### 2.13 Statistical analysis

Results were presented as mean ± SEM from at least three independent experiments. Analyses were performed with Prism GraphPad 8.0.1 (GraphPad Software). All data sets were tested for Gaussian distribution. The unpaired *t* test and one‐way ANOVA were used to analyse statistical significance. A two‐tailed *P* < .05 was statistical significance.

## 3 RESULTS

### 3.1 PBMT could protect against atherosclerosis

**FIGURE 1:**
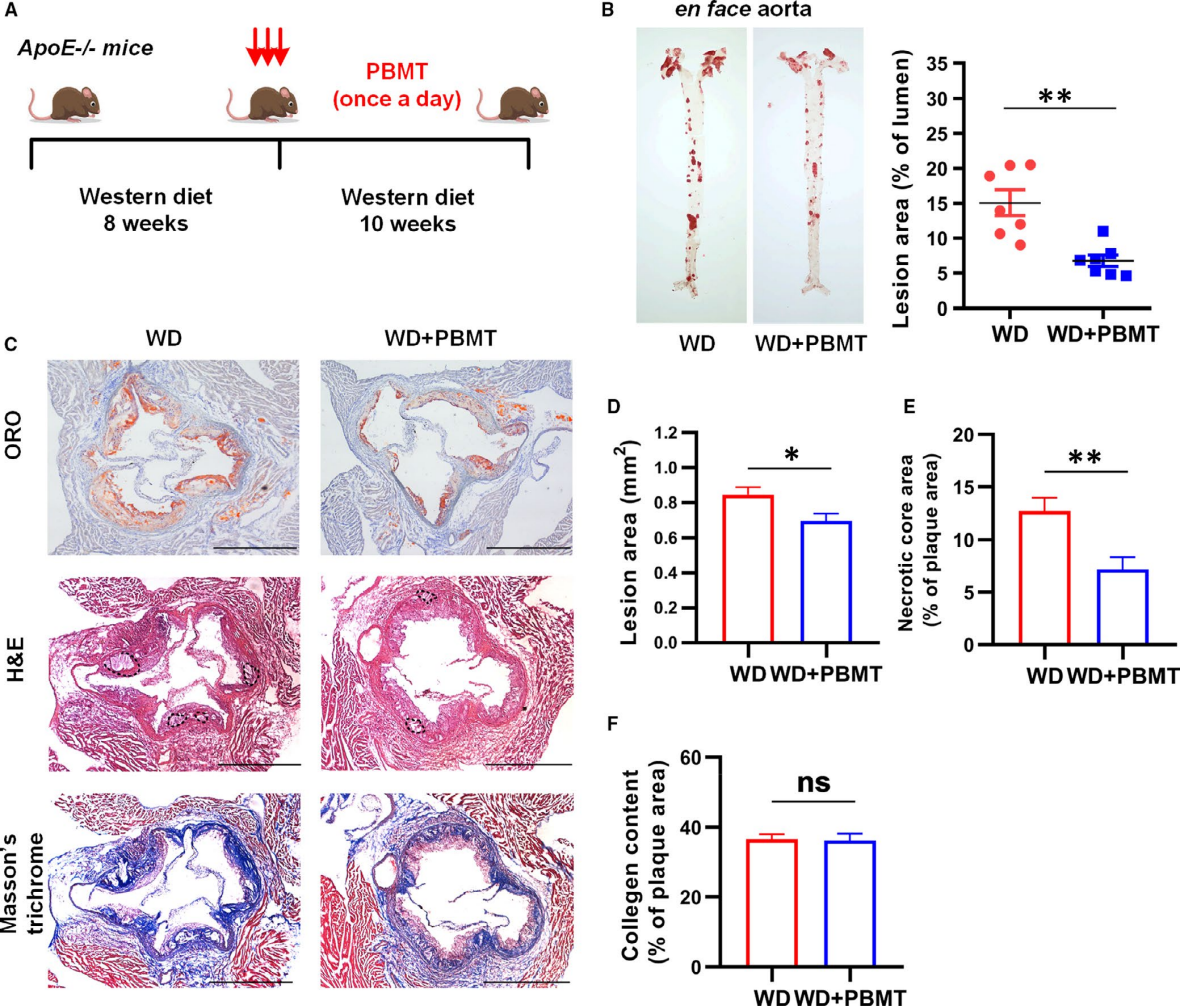
Photobiomodulation therapy (PBMT) could protect against atherosclerosis. A, Schematic diagram. *Apolipoprotein E* (*ApoE*)^−/−^ mice fed with a western diet (WD) for 18 wk and treated with PBMT in the last 10 wk. B, Representative ORO staining and quantitative analysis of atherosclerosis lesion area in *ApoE* ^−/−^ mice aortas (n = 7). C‐F, Representative ORO, H&E and Masson's trichrome staining and quantitative analysis of the lesion area D, necrotic core E, and collagen content F, in the aortic sinus. Scale bars, 400 μm (n = 8). Data are shown as mean ± SEM, WD vs. WD + PBMT, unpaired two‐tailed Student's *t* test. *P < .05, **P < .01, *ns* = not significant

Studies had reported that PBMT improved blood lipids and haemorheology of patients with coronary heart disease or cerebral infarction;^24^ however, the effect and mechanism of PBMT on atherosclerosis need to be explored. First, a model of atherosclerosis had been established on *ApoE* ^−/−^ mice following a WD, and then, the model mice were treated with PBMT for 10 weeks (Figure 1A). We removed the aorta of the mice and used ORO staining to detect atherosclerosis lesion. *En face* analysis revealed that the average atherosclerosis lesion area decreased 8.306 ± 2.012% after PBMT (*P* < .05). (Figure 1B). Since the aortic sinus is prone to atherosclerosis, we also compared atherosclerosis lesion, necrosis core area and collagen content in these areas (Figure 1C). H&E staining and ORO staining of cross sections revealed that significantly smaller lesion and necrosis core areas occurred in WD + PBMT mice (Figure 1D,E). However, there was no significant change in collagen content, suggesting that PBMT could stabilize the plaque by reducing the size of the necrotic core instead of affecting the collagen content (Figure 1F). The above results indicate that PBMT could reduce the area of atherosclerotic plaque, and thus have a certain protective effect on atherosclerosis.

### 3.2 PBMT could improve plasma lipoprotein levels and inhibit foam cells formation

**FIGURE 2:**
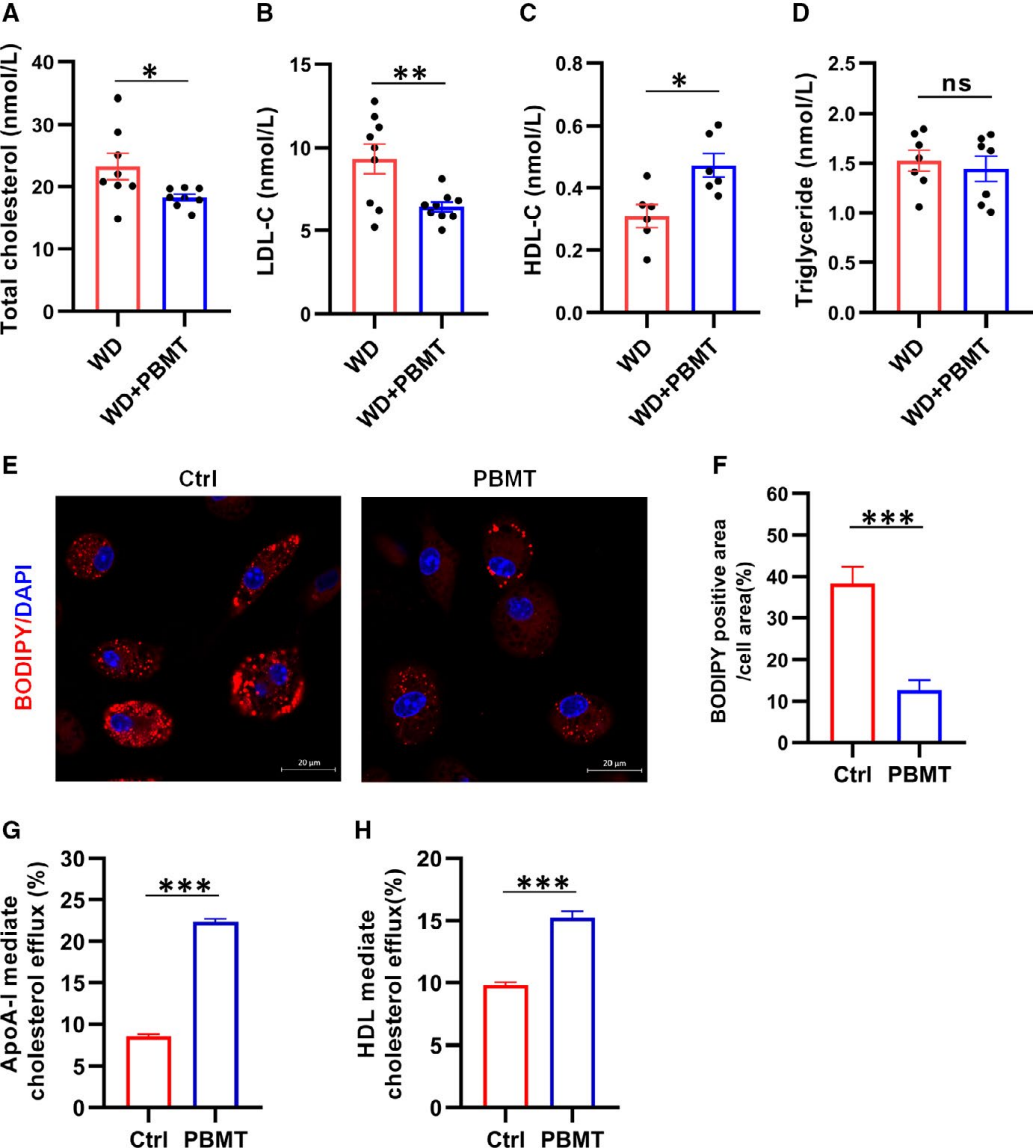
Photobiomodulation therapy (PBMT) could improve plasma lipoprotein levels and inhibit foam cells formation. A‐D, Representative total cholesterol level, LDL cholesterol level (LDL‐C), high‐density lipoprotein cholesterol level (HDL‐C) and triglyceride level in the plasma of *Apolipoprotein E* (*ApoE*)^−/−^ mice after 18 wk on western diet (WD; n = 7‐9). E, F, BODIPY staining E, and quantitative statistics F, showed the lipid content in primary mouse peritoneal macrophages after treated with PBMT. Scale bars, 20 μm (n = 6). G, H, apolipoprotein A‐I (ApoA‐I) G, and High‐density lipoprotein H (HDL H; respect of the ATP‐binding cassette transporters A1 [ABCA1] and ATP‐binding cassette transporter G1 [ABCG1])‐mediated cholesterol efflux after treated with PBMT (n = 7‐12). Data are shown as mean ± SEM, WD vs WD + PBMT, unpaired two‐tailed Student's *t* test. *P < .05, **P < .01, ***P < .001, *ns* = not significant

Hyperlipidaemia is regarded as an important risk factor for atherosclerosis. Therefore, we next examined the effect of PBMT on plasma lipoprotein levels. Results revealed that total cholesterol and LDL‐C levels in WD + PBMT mice respectively reduced 5.0 ± 2.2 nmol/L (*P* < .05) and 2.9 ± 0.94 nmol/L (*P* < .01) compared with WD mice. HDL‐C level in mice after processing with PBMT increased from 0.309 ± 0.037 nmol/L to 0.472 ± 0.038 nmol/L (*P* < .05), but had no difference in triglyceride level compared with WD mice (Figure 2A‐D). Foam cells formation is a hallmark of atherosclerotic lesions. Promoting the RCT process can effectively promote the formation of HDL and reduce the accumulation of intracellular lipids in cells. To test whether PBMT inhibits foam cells formation, we load mouse primary peritoneal macrophages with oxLDL to simulate a foam cell model. First, we determined the amount of lipids in macrophages after treating with PBMT to confirmed optimum concentration of oxLDL and dose of PBMT (Figures S1 and S2). BODIPY staining showed that cells treated with PBMT had less lipid content than control (Figure 2E,F), suggesting that PBMT could reduce lipids accumulation in macrophages. ABCA1 and ABCG1‐mediated cholesterol efflux is the rate‐limiting step of RCT and considered to be an effective method to suppress foam cells formation. Then, we detected ApoA‐I and HDL‐mediated cholesterol efflux using 22‐NBD cholesterol (which represent ABCA1 and ABCG1‐mediated cholesterol efflux, respectively). Results suggested that the ABCA1 and ABCG1‐medicated cholesterol efflux in macrophages were increased about 13.9% and 5%, respectively, after PBMT (Figure 2G,H). A more significant increase was observed in ABCA1‐medicated cholesterol efflux; therefore, in the follow‐up experiments, we will focus on the impact of PBMT on ABCA1. We draw conclusions from the above experiments that PBMT could improve plasma lipoprotein levels and inhibit foam cells formation.

### 3.3 PBMT could promote ABCA1 expression and ABCA1‐dependent cholesterol efflux

**FIGURE 3:**
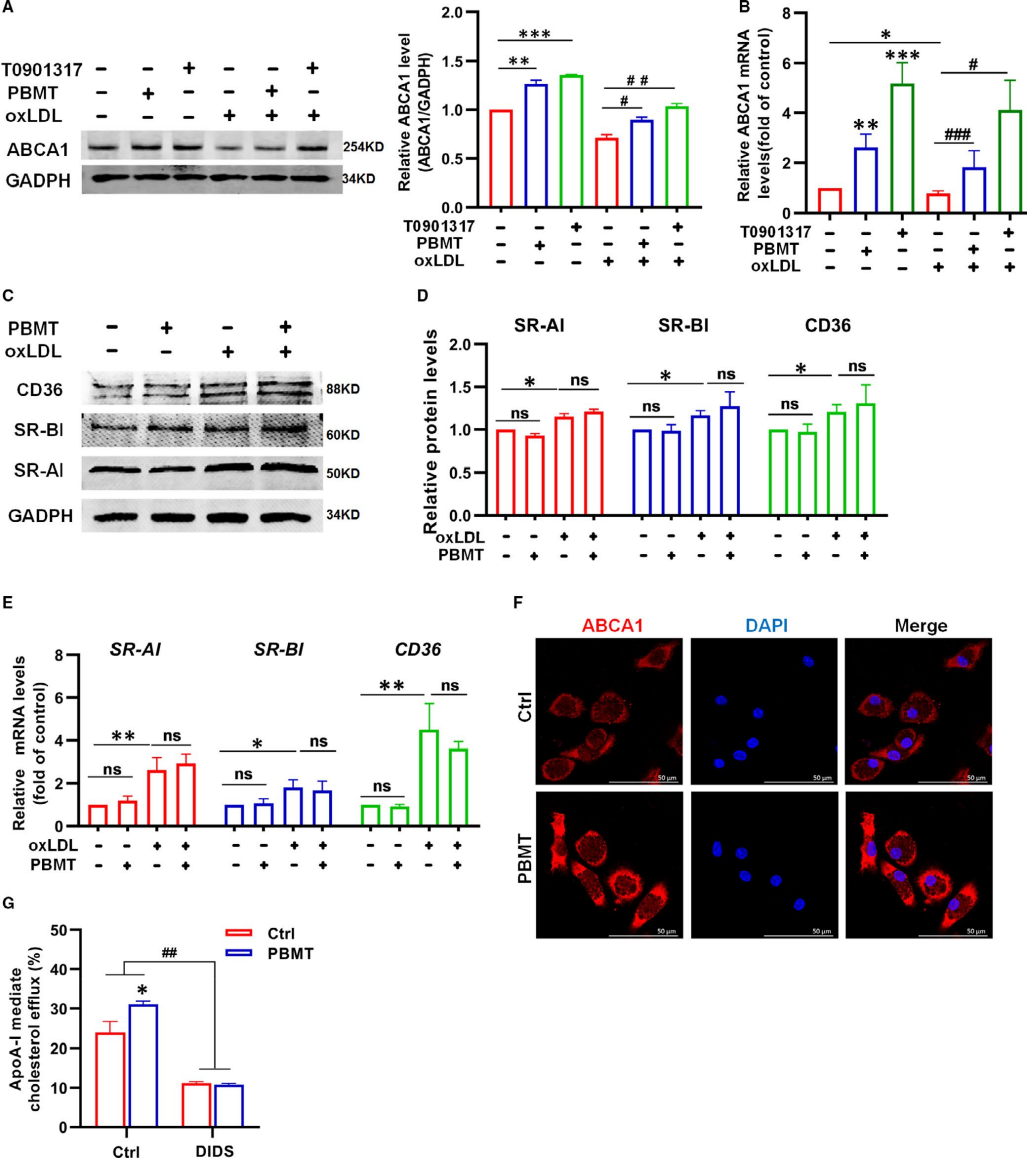
Photobiomodulation therapy (PBMT) could promote ATP‐binding cassette transporters A1 (ABCA1) expression and ABCA1‐dependent cholesterol efflux. A, Western blot (WB) analysis and quantification of ABCA1 expression in primary mouse peritoneal macrophages or lipid‐loaded macrophages after treated with PBMT (n = 6). B, qPCR analysis of ABCA1 expression in primary mouse peritoneal macrophages or lipid‐loaded macrophages after treated with PBMT or positive control T0901317 (n = 8). C, D, WB analysis and quantification of SR‐AI, SR‐BI and CD36 expression in primary mouse peritoneal macrophages or lipid‐loaded macrophages after treated with PBMT (n = 4). E, qPCR analysis of SR‐AI, SR‐BI and CD36 expression in primary mouse peritoneal macrophages or lipid‐loaded macrophages after treated with PBMT (n = 8). F, Representative ABCA1 staining of mouse peritoneal macrophages showing ABCA1 expression after treated with PBMT. Scale bars, 50 μm. G, apolipoprotein A‐I (ApoA‐I) mediated cholesterol efflux after treat with PBMT or DIDS (n = 6). Data are shown as mean ± SEM, control vs PBMT, unpaired two‐tailed Student's *t* test or one‐way ANOVA. *P < .05, **P < .01, ***P < .001, ^#^ *P* < .05, ^##^ *P* < .01, ^###^ *P* < .001, *ns* = not significant

The front experiments confirmed that PBMT could significantly promote ABCA1‐medicated cholesterol efflux from macrophages and inhibit foam cells formation. Thus, we next examined the effect of PBMT on ABCA1. WB and qPCR experiments results suggested that a significant increase of ABCA1 was observed in oxLDL‐loaded and non‐loaded cells after treating with PBMT (Figure 3A,B). We also detected the expression of SRs associated with foam cells formation after PBMT treatment by WB and qPCR experiments. Data revealed that the expression of SR‐AI, SR‐BI and CD36 in oxLDL‐loaded cells were significantly higher than oxLDL non‐loaded cells, and PBMT did not affect the expression of SR‐AI, SR‐BI and CD36 (Figure 3C‐E), indicating that PBMT treatment would not promote excessive phagocytosis of lipoproteins by macrophages. Immunofluorescence staining showed that the ABCA1 content on the membrane of macrophages was significantly higher than control group after PBMT treatment (Figure 3F). These findings indicated that PBMT could up‐regulate the expression of ABCA1 to promote the cholesterol handle in macrophages rather than affecting lipoprotein uptake. To further confirm that cholesterol efflux promoted by PBMT was ABCA1‐dependent, DIDS, specific inhibitor of ABCA1, was added. The result verified that PBMT could promote macrophages ABCA1‐medicated cholesterol efflux and the increase of cholesterol efflux was inhibited in the presence of DIDS (Figure 3G). Overall, we conclude that PBMT inhibits foam cells formation by up‐regulating ABCA1 expression to promote ABCA1‐medicated cholesterol efflux.

### 3.4 ABCA1 was up‐regulated by PBMT in *ApoE* ^−/−^ mice aortic tissue and peritoneal macrophages

**FIGURE 4:**
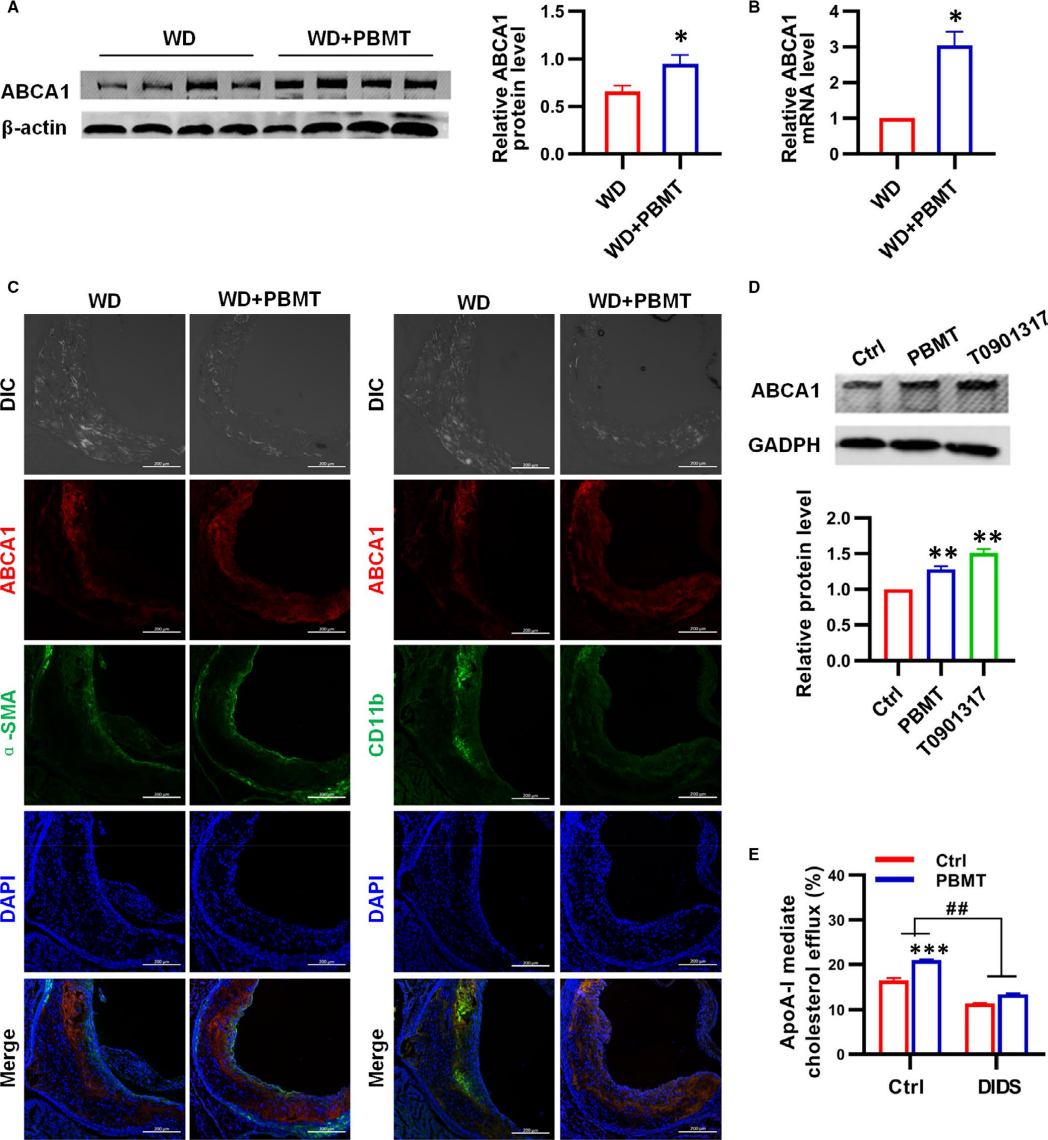
ATP‐binding cassette transporters A1 (ABCA1) was up‐regulated by Photobiomodulation therapy (PBMT) in *Apolipoprotein E* (*ApoE*)^−/−^ mice aortic tissue and peritoneal macrophages. A, Western blot (WB) analysis and quantification of ABCA1 expression in *ApoE* ^−/−^ mice aortic after treated with PBMT (n = 8). B, qPCR analysis of ABCA1 expression in *ApoE* ^−/−^ mice aortic after treated with PBMT (n = 5). C, Representative ABCA1, α‐SMA (smooth muscle cell) and CD11b (macrophage) staining in the aortic sinus after treated with PBMT. Scale bars, 200 μm. D, WB analysis and quantification of ABCA1 expression in *ApoE* ^−/−^ mice peritoneal macrophages (n = 4). E, apolipoprotein A‐I (ApoA‐I)‐mediated cholesterol efflux after treat with PBMT or DIDS in *ApoE* ^−/−^ mice peritoneal macrophages (n = 6). Data are shown as mean ± SEM, control vs PBMT, unpaired two‐tailed Student's *t* test. *P < .05, **P < .01, ***P < .001, ^##^ *P* < .01, *ns* = not significant

The above results indicated that PBMT inhibited intracellular lipid accumulation by promoting ABCA1‐mediated cholesterol efflux *in vitro*. However, did PBMT also inhibit atherosclerosis development through this pathway *in vivo*? The following aim was to determine whether remission of atherosclerosis by PBMT resulted from an up‐regulation of ABCA1 in vivo. The WB and qPCR assay of *ApoE* ^−/−^ mice aortic tissue lysates revealed that cholesterol transporter ABCA1 was up‐regulated in WD + PBMT mice (Figure 4A,B). We carried immunofluorescence staining and found that the aortic sinus plaque area of WD + PBMT mice was smaller than control group, and the number of macrophages in the plaques was less than the control group. We also observed that ABCA1 is mainly expressed on macrophages, and a small amount is expressed on smooth muscle cells (SMC) in atherosclerotic plaques. More importantly, PBMT treatment could increase the expression of ABCA1 in mouse aortic sinus plaque (Figure 4C). The expression of ABCA1 and ABCA1‐mediated cholesterol efflux was also examined in peritoneal macrophages from *ApoE* ^−/−^ mice bearing atherosclerotic lesions. Results suggested that the expression level of ABCA1 after PBMT was 1.3 times of those in the control group (*P* < .01; Figure 4D). And after PBMT treatment, cholesterol efflux mediated by ABCA1 was significantly higher than the control group (Figure 4E). These findings indicated that PBMT could up‐regulate ABCA1 expression *in vivo* to inhibit atherosclerosis development.

### 3.5 Activation of PI3K/PKCζ /SP1 signalling cascade by PBMT promoted ABCA1 expression and cholesterol efflux

**FIGURE 5:**
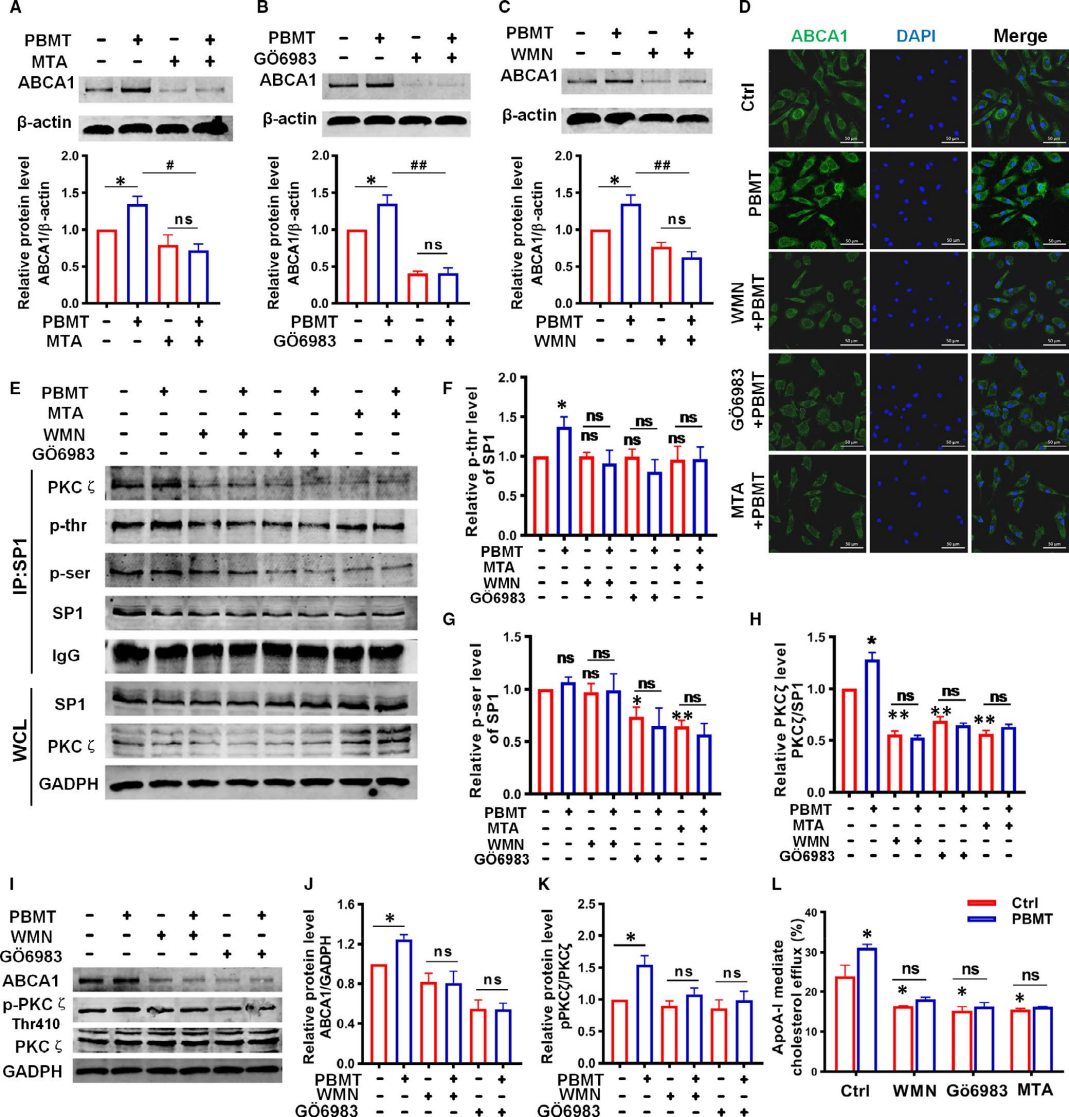
Activation of phosphatidylinositol 3‐kinases/protein kinase C zeta/specificity protein 1 (PI3K/PKCζ/SP1) signalling cascade by Photobiomodulation therapy (PBMT) promoted ATP‐binding cassette transporters A1 (ABCA1) expression and cholesterol efflux. A‐C, Western blot (WB) analysis of ABCA1 expression in primary mouse peritoneal macrophages with different treatments: Mithramycin A (MTA, 200 nmol/L) A, GÖ6983 (4 μmol/L) B, and wortmannin (WMN, 200 nmol/L) C, (n = 4). D, Representative ABCA1 staining in primary mouse peritoneal macrophages with different treatments. Scale bars, 50 μm. E‐H, Immunoprecipitation (IP) of SP1 and WB E, and statistical analysis of SP1 threonine F, serine G, residues phosphorylation and PKCζ bound to SP1 H, after treated with or without PBMT, WMN, GÖ6983 and MTA (n = 3). I‐K, WB I, and statistical analysis of PKCζ phosphorylation J, and ABCA1 expression K, after treated with PBMT, WMN and GÖ6983 (n = 3). L, Apolipoprotein A‐I (ApoA‐I)‐mediated cholesterol efflux after treat with or without PBMT, WMN, GÖ6983 and MTA (n = 6). Data are shown as mean ± SEM, one‐way ANOVA *P < .05, **P < .01, ^#^ *P* < .05, ^##^ *P* < .01, *ns* = not significant

The former results demonstrated that PBMT up‐regulated the ABCA1 expression in both mouse aortic and peritoneal macrophages, then we did a series of experiments to explore the underlying mechanism. Some studies have reported that SP1 is related to ABCA1 expression, and previous work also found the phosphorylation of SP1 by PBMT promoted its downstream gene transcription.^30^, ^31^, ^32^ To evaluate the role of SP1 in PBMT‐induced ABCA1 expression, specific SP1 inhibitor, MTA, was used and the WB showed that inhibitions of SP1 notably suppressed PBMT‐induced ABCA1 upexpression (Figure 5A). SP1 is a class of transcription factors whose activity is regulated by post‐transcription. AKT and PKCζ, which have been reported involved in regulating the activity of SP1, also associated with the expression of ABCA1. Therefore, we subsequently conducted a series of WB experiments to explore whether these proteins are involved in the process of PBMT up‐regulating ABCA1 expression. As shown in Figure 5B, PBMT‐induced ABCA1 expression was inhibited when PKCζ inhibitors were added but had no significant difference in the group which AKT was inhibited (Figure S3). It has been reported that the activity of PKCζ was regulated by PI3K.^33^, ^34^ We next determined whether PI3K was required in PBMT‐induced ABCA1 expression and the significant reduction in ABCA1 protein level after PBMT was observed when PI3K was inhibited in the WB analysis (Figure 5C). We observed similar results in immunofluorescence staining of ABCA1 (Figure 5D). Under these results, we concluded that PBMT‐induced ABCA1 expression was regulated by SP1, PKCζ and PI3K. Next, we further found out through the immunoprecipitation (IP) and WB analysis that PBMT increased the phosphorylation of threonine but not the serine sites in SP1 and the threonine site phosphorylation level was decreased when PI3K, PKCζ and SP1 were inhibited, respectively (Figure 5E‐G). The total protein of SP1 and PKCζ did not change significantly after different treatments (Figure 5E, Figure S4). Another result showed that the combination of PKCζ and SP1 was increased after treating with PBMT, and the increase was weakened in the presence of the PI3K, PKCζ and SP1 inhibitors. (Figure 5E,H), suggesting that PBMT could promote PKCζ bound to SP1 and subsequently activate SP1. Additionally, we found that the phosphorylation of PKCζ was increased after treating with PBMT, and the increase was inhibited when WMN and GÖ6983 were added, the change of ABCA1 expression was on the same trajectory (Figure 5I‐K). From the above, those results suggested that activation of PI3K/PKCζ/SP1 signalling pathway by PBMT promoted ABCA1 expression. Finally, we explored whether PI3K, PKCζ and SP1 are involved in the regulation of cholesterol efflux promoted by PBMT. The inhibitors of PI3K, PKCζ and SP1 were employed again to observe the changes of cholesterol efflux and results showed that they weakened the increase of cholesterol efflux induced by PBMT (Figure 5L). In summary, activation of PI3K/PKCζ/SP1 signalling cascade by PBMT promoted ABCA1 expression and ABCA1‐mediated cholesterol efflux in macrophages.

## 4 DISCUSSION

It is well known that atherosclerosis is an immune system‐mediated chronic inflammatory disease. Cholesterol‐rich macrophages formation and retention in arterial vasculature exacerbates the disease and promotes the development of vulnerable plaques, causing acute clinical events.^35^, ^36^ Therefore, strategies aimed to diminish macrophage foam cells formation can slow the lesion progression.^37^ The imbalance of cholesterol influx and efflux makes cholesterol esters accumulate in macrophages to form the foam cells. It is well‐established that cholesterol transporters‐mediated cholesterol efflux and RCT are major mechanisms for the removal of cellular cholesterol. Here, we tested the hypothesis that PBMT had beneficial therapeutic effects on WD‐induced atherosclerosis. The results from the lipid and histomorphology studies combined with in vivo studies in atherosclerotic mice suggested that PBMT exerted its potent effect by promoting macrophage cholesterol efflux to inhibit the formation of foam cells in atherosclerotic plaques.

In atherosclerosis, macrophage‐dependent cholesterol handling is deregulated. Pro‐inflammatory stimulus up‐regulates SRs expression in macrophages, in contrast, ABCA1 and ABCG1 expression is decreased, further exacerbating the accumulation of intracellular cholesterol and promoting the production of foam cells.^38^ As reported, ABCA1 and ABCG1 work together in mediating cholesterol efflux to HDL. What we found in our study was that PBMT significantly promoted ABCA1‐medicated cholesterol efflux. The importance of ABCA1 transporter for cholesterol efflux from macrophages has been demonstrated in many studies. Indeed, the ability to up‐regulate ABCA1 expression may be a key determinant of macrophages cholesterol efflux. We have found that treating with PBMT promoted ABCA1‐mediated cholesterol efflux in macrophages by increasing ABCA1 mRNA and protein levels. Evidence confirmed that the effect of PBMT on cholesterol efflux was suppressed when ABCA1 expression was diminished.

Photobiomodulation therapy or low‐level light/laser therapy was accidentally discovered in 1967 and has been used clinically for over 20 years in analgesia, anti‐inflammatory and wound healing, with a long safety record. Liu et al apply PBMT to clinical treatment of vascular diseases, and their research showed that total cholesterol level and LDL‐C decreased significantly, and HDL‐C increased significantly. Researchers deemed that PBMT might be a systemic effect and the mesenchymal stem cells/marrow stromal cells or blood cells might be the targets, so they carried the trials through intranasal low‐intensity GaInP/AlGaInP diode 650 nm laser.^24^ We also consider that the effect of PBMT is systematic and blood cells are the target of PBMT, because of rich capillaries on the inner surface of the nasal cavity. Tulebaev found that PBMT irradiation through nasal cavity modified all responses of T lymphocytes and they observed a significant increase of T lymphocytes and a higher capacity of T cells to form the migration inhibition factor.^39^ Other researches also came to a similar conclusion.^40^, ^41^ Therefore, in our experiment, animals received laser irradiation through the abdomen and chest to get a systemic effect through peripheral blood. We did observe some changes in mouse peritoneal macrophages, which prove our guess.

There are limitations in our study. First, given the laser applied to the abdomen and chest, maybe there were some impacts on gut or liver. However, we just observed the changes of some indicators without in‐depth discussion. Second, liver X receptors (LXR), retinoic acid receptors and peroxisome proliferator‐activated receptors (PPARs) along with their coactivator have been reported to amplify ABCA1 expression,^42^, ^43^, ^44^ changes in these factors were not detected in our experiments. Third, in addition to serving as a transcription factor, SP1 can also act as a transcription coactivator to promote the binding of LXR‐RXR to the promoter region of ABCA1 gene.^45^, ^46^ Our research did not explore this aspect and all of these will be verified in our future experiments.

Overall, the current investigation demonstrates that PBMT, a non‐invasive treatment, can ameliorate WD‐induced atherosclerosis through promoting the ABCA1‐medicated cholesterol efflux to inhibit foam cells formation. PKCζ‐dependent phosphorylation of SP1 threonine sites is critical for up‐regulating ABCA1 expression by PBMT. Our studies provide a proof that therapies aimed at increasing the removal of macrophage cholesterol by PBMT could be a valid strategy for treating atherosclerosis. Some of the key advantages of PBMT are its economy and safety, because it can be used with commercially available light‐emitting devices to transfer energy within a safe range for humans. Therefore, PBMT might be a meaningful therapeutic tool for CVD.

## CONFLICT OF INTEREST

None.

## AUTHOR CONTRIBUTIONS

**Qianxia Yin:** Data curation (lead); Investigation (lead); Writing‐original draft (lead). **Haocai Chang:** Formal analysis (equal); Methodology (equal); Writing‐review & editing (lead). **Qi Shen:** Formal analysis (supporting); Writing‐review & editing (lead). **Da Xing:** Conceptualization (lead); Funding acquisition (lead); Project administration (lead).

## Supporting information

Supplementary Material

## Data Availability

The data sets used and/or analysed during the current study are available from the corresponding author on reasonable request.

## References

[1] Libby P , Ridker PM , Hansson GK . Progress and challenges in translating the biology of atherosclerosis. Nature. 2011;473:317‐325. 21593864 10.1038/nature10146

[2] Moore KJ , Tabas I . Macrophages in the pathogenesis of atherosclerosis. Cell. 2011;145:341‐355. 21529710 10.1016/j.cell.2011.04.005 PMC3111065

[3] Moore KJ , Sheedy FJ , Fisher EA . Macrophages in atherosclerosis: a dynamic balance. Nat Rev Immunol. 2013;13:709‐721. 23995626 10.1038/nri3520 PMC4357520

[4] Navab M , Reddy ST , Van Lenten BJ , Fogelman AM . HDL and cardiovascular disease: atherogenic and atheroprotective mechanisms. Nat Rev Cardiol. 2011;8:222‐232. 21304474 10.1038/nrcardio.2010.222

[5] Wang D , Yang Y , Lei Y , et al. Targeting foam cell formation in atherosclerosis: therapeutic potential of natural products. Pharmacol Rev. 2019;71:596‐670. 31554644 10.1124/pr.118.017178

[6] Westerterp M , Fotakis P , Ouimet M , et al. Cholesterol efflux pathways suppress inflammasome activation, NETosis, and atherogenesis. Circulation. 2018;138:898‐912. 29588315 10.1161/CIRCULATIONAHA.117.032636 PMC6160368

[7] Wang N , Tall AR . Regulation and mechanisms of ATP‐binding cassette transporter A1‐mediated cellular cholesterol efflux. Arterioscler Thromb Vasc Biol. 2003;23:1178‐1184. 12738681 10.1161/01.ATV.0000075912.83860.26

[8] Oram JF . Molecular basis of cholesterol homeostasis: lessons from Tangier disease and ABCA1. Trends Mol Med. 2002;8:168‐173. 11927274 10.1016/s1471-4914(02)02289-x

[9] Marcil M , Brooks‐Wilson A , Clee SM , et al. Mutations in the ABC1 gene in familial HDL deficiency with defective cholesterol efflux. Lancet. 1999;354:1341‐1346. 10533863 10.1016/s0140-6736(99)07026-9

[10] Singaraja RR , Fievet C , Castro G , et al. Increased ABCA1 activity protects against atherosclerosis. J Clin Invest. 2002;110:35‐42. 12093886 10.1172/JCI15748 PMC151034

[11] Antunes HS , de Azevedo AM , Bouzas LFD , et al. Low‐power laser in the prevention of induced oral mucositis in bone marrow transplantation patients: a randomized trial. Blood. 2007;109:2250‐2255. 17053058 10.1182/blood-2006-07-035022

[12] Zivin JA , Albers GW , Bornstein N , et al. Effectiveness and safety of transcranial laser therapy for acute ischemic stroke. Stroke. 2009;40:1359‐1364. 19233936 10.1161/STROKEAHA.109.547547

[13] Silveira PCL , Silva LA , Freitas TP , Latini A , Pinho RA . Effects of low‐power laser irradiation (LPLI) at different wavelengths and doses on oxidative stress and fibrogenesis parameters in an animal model of wound healing. Laser Med Sci. 2011;26:125‐131. 10.1007/s10103-010-0839-0 20865435

[14] Chen CH , Hung HS , Hsu SH . Low‐energy laser irradiation increases endothelial cell proliferation, migration, and eNOS gene expression possibly via PI3K signal pathway. Laser Surg Med. 2008;40:46‐54. 10.1002/lsm.20589 18220263

[15] Feng J , Zhang YJ , Xing D . Low‐power laser irradiation (LPLI) promotes VEGF expression and vascular endothelial cell proliferation through the activation of ERK/Sp1 pathway. Cell Signal. 2012;24:1116‐1125. 22326662 10.1016/j.cellsig.2012.01.013

[16] Arany PR , Cho A , Hunt TD , et al. Photoactivation of endogenous latent transforming growth factor‐beta1 directs dental stem cell differentiation for regeneration. Sci Transl Med. 2014;6:238ra269. 10.1126/scitranslmed.3008234 PMC4113395 24871130

[17] Tuby H , Maltz L , Oron U . Induction of autologous mesenchymal stem cells in the bone marrow by low‐level laser therapy has profound beneficial effects on the infarcted rat heart. Lasers Surg Med. 2011;43:401‐409. 21674545 10.1002/lsm.21063

[18] Gavish L , Asher Y , Becker Y , Kleinman Y . Low level laser irradiation stimulates mitochondrial membrane potential and disperses subnuclear promyelocytic leukemia protein. Lasers Surg Med. 2004;35:369‐376. 15611960 10.1002/lsm.20108

[19] Pastore D , Greco M , Passarella S . Specific helium‐neon laser sensitivity of the purified cytochrome c oxidase. Int J Radiat Biol. 2000;76:863‐870. 10902741 10.1080/09553000050029020

[20] Zhang Q , Dong T , Li P , Wu MX . Noninvasive low‐level laser therapy for thrombocytopenia. Sci Transl Med. 2016;8:349ra101. 10.1126/scitranslmed.aaf4964 PMC7392149 27464749

[21] Meng C , He Z , Xing D . Low‐level laser therapy rescues dendrite atrophy via upregulating BDNF expression: implications for Alzheimer's disease. J Neurosci. 2013;33:13505‐13517. 23946409 10.1523/JNEUROSCI.0918-13.2013 PMC6705158

[22] Kipshidze N , Nikolaychik V , Keelan MH , et al. Low‐power helium : neon laser irradiation enhances production of vascular endothelial growth factor and promotes growth of endothelial cells in vitro. Laser Surg Med. 2001;28:355‐364. 10.1002/lsm.1062 11344517

[23] Tuby H , Maltz L , Oron U . Modulations of VEGF and iNOS in the rat heart by low level laser therapy are associated with cardioprotection and enhanced angiogenesis. Laser Surg Med. 2006;38:682‐688. 10.1002/lsm.20377 16800001

[24] Liu TCY , Cheng L , Su WJ , et al. Double‐blind, and placebo‐controlled clinic report of intranasal low‐intensity laser therapy on vascular diseases. Int J Photoenergy. 2012;2012:1‐5.

[25] Gavish L , Rubinstein C , Berlatzky Y , et al. Low level laser arrests abdominal aortic aneurysm by collagen matrix reinforcement in apolipoprotein E‐deficient mice. Laser Surg Med. 2012;44:664‐674. 10.1002/lsm.22068 22911625

[26] Hartley CJ , Reddy AK , Madala S , et al. Hemodynamic changes in apolipoprotein E‐knockout mice. Am J Physiol Heart Circ Physiol. 2000;279:H2326‐H2334. 11045969 10.1152/ajpheart.2000.279.5.H2326

[27] Ma Z , Choudhury A , Kang SA , Monestier M , Cohen PL , Eisenberg RA . Accelerated atherosclerosis in ApoE deficient lupus mouse models. Clin Immunol. 2008;127:168‐175. 18325838 10.1016/j.clim.2008.01.002 PMC2464279

[28] Brown DL . Practical stereology applications for the pathologist. Vet Pathol. 2017;54:358‐368. 28438109 10.1177/0300985817695781

[29] Muhlfeld C , Wrede C , Knudsen L , Buchacker T , Ochs M , Grothausmann R . Recent developments in 3‐D reconstruction and stereology to study the pulmonary vasculature. Am J Physiol Lung Cell Mol Physiol. 2018;315:L173‐L183. 29644892 10.1152/ajplung.00541.2017

[30] Chen XP , Zhao YF , Guo ZM , Zhou LC , Okoro EU , Yang H . Transcriptional regulation of ATP‐binding cassette transporter A1 expression by a novel signaling pathway. J Biol Chem. 2011;286:8917‐8923. 21257755 10.1074/jbc.M110.214429 PMC3058999

[31] Chen XP , Guo ZM , Okoro EU , et al. Up‐regulation of ATP binding cassette transporter A1 expression by very low density lipoprotein receptor and apolipoprotein E receptor 2. J Biol Chem. 2012;287:3751‐3759. 22170052 10.1074/jbc.M111.310888 PMC3281687

[32] Schmitz G , Langmann T . Transcriptional regulatory networks in lipid metabolism control ABCA1 expression. BBA‐Mol Cell Biol L. 2005;1735:1‐19. 10.1016/j.bbalip.2005.04.004 15922656

[33] Nakanishi H , Brewer KA , Exton JH . Activation of the zeta isozyme of protein kinase C by phosphatidylinositol 3,4,5‐trisphosphate. J Biol Chem. 1993;268:13‐16. 8380153

[34] Herrera‐Velit P , Knutson KL , Reiner NE . Phosphatidylinositol 3‐kinase‐dependent activation of protein kinase C‐zeta in bacterial lipopolysaccharide‐treated human monocytes. J Biol Chem. 1997;272:16445‐16452. 9195953 10.1074/jbc.272.26.16445

[35] Back M , Hansson GK . Anti‐inflammatory therapies for atherosclerosis. Nat Rev Cardiol. 2015;12:199‐211. 25666404 10.1038/nrcardio.2015.5

[36] Witztum JL , Lichtman AH . The influence of innate and adaptive immune responses on atherosclerosis. Annu Rev Pathol. 2014;9:73‐102. 23937439 10.1146/annurev-pathol-020712-163936 PMC3988528

[37] Randolph GJ . Mechanisms that regulate macrophage burden in atherosclerosis. Circ Res. 2014;114:1757‐1771. 24855200 10.1161/CIRCRESAHA.114.301174 PMC4059102

[38] Hutchins PM , Heinecke JW . Cholesterol efflux capacity, macrophage reverse cholesterol transport and cardioprotective HDL. Curr Opin Lipidol. 2015;26:388‐393. 26270810 10.1097/MOL.0000000000000209 PMC4617325

[39] Tulebaev RK , Sadykov SHB , Romanov VA , Khalitova G . Indicators of the activity of the immune system during laser therapy of vasomotor rhinitis. Vestn Otorinolaringol. 1989;1:46‐49. 2785311

[40] Kruchinina I , Feniksova LV , Rybalkin SV , Pekli FF . Therapeutic effect of helium‐neon laser on microcirculation of nasal mucosa in children with acute and chronic maxillary sinusitis as measured by conjunctival biomicroscopy. Vestn Otorinolaringol. 1991;3:26‐30. 1862594

[41] Shevrygin BV , Rybalkin SV , Pekli FF , Feniksova LV . Correction of microcirculatory disorders with low‐energy laser radiation in children with vasomotor rhinitis. Vestn Otorinolaringol. 2000;2:31‐33. 10771608

[42] Chawla A , Boisvert WA , Lee CH , et al. A PPAR gamma‐LXR‐ABCA1 pathway in macrophages is involved in cholesterol efflux and atherogenesis. Mol Cell. 2001;7:161‐171. 11172721 10.1016/s1097-2765(01)00164-2

[43] Chinetti G , Lestavel S , Bocher V , et al. PPAR‐alpha and PPAR‐gamma activators induce cholesterol removal from human macrophage foam cells through stimulation of the ABCA1 pathway. Nat Med. 2001;7:53‐58. 11135616 10.1038/83348

[44] Uehara Y , Miura S , von Eckardstein A , et al. Unsaturated fatty acids suppress the expression of the ATP‐binding cassette transporter G1 (ABCG1) and ABCA1 genes via an LXR/RXR responsive element. Atherosclerosis. 2007;191:11‐21. 16730733 10.1016/j.atherosclerosis.2006.04.018

[45] Langmann T , Porsch‐Ozcurumez M , Heimerl S , et al. Identification of sterol‐independent regulatory elements in the human ATP‐binding cassette transporter A1 promoter ‐ Role of Sp1/3, E‐box binding factors, and an oncostatin M‐responsive element. J Biol Chem. 2002;277:14443‐14450. 11839742 10.1074/jbc.M110270200

[46] Yang XP , Freeman LA , Knapper CL , et al. The E‐box motif in the proximal ABCA1 promoter mediates transcriptional repression of the ABCA1 gene. J Lipid Res. 2002;43:297‐306. 11861672

