# Supplementary material for: Photobiomodulation therapy promotes the ATP‐binding cassette transporter A1‐dependent cholesterol efflux in macrophage to ameliorate atherosclerosis
Source: J Cell Mol Med. 2021 May 5;25(11):5238–49. doi: 10.1111/jcmm.16531 (PMC8178257; doi:10.1111/jcmm.16531)
Supplement: Supplementary file 1 — Supplementary Material [file JCMM-25-5238-s001.docx]

**Supporting information**

**Photobiomodulation therapy promotes the ABCA1-dependent cholesterol efflux in macrophage to** **ameliorate atherosclerosis**

Qianxia Yin^1,2^, Haocai Chang^1,2^, Qi Shen^1,2^, Da Xing^1,2^*

^1^ MOE Key Laboratory of Laser Life Science & Institute of Laser Life Science, South China Normal University, Guangzhou 510631, China

^2^ College of Biophotonics, South China Normal University, Guangzhou 510631, China

**Address correspondence to:**

Da Xing, MOE Key Laboratory of Laser Life Science & Institute of Laser Life Science

College of Biophotonics, South China Normal University

Guangzhou 510631, China

E-mail: [xingda@scnu.edu.cn](mailto:xingda@scnu.edu.cn)

Tel: +86-20-85210089

**Supplementary Figure 1**

**
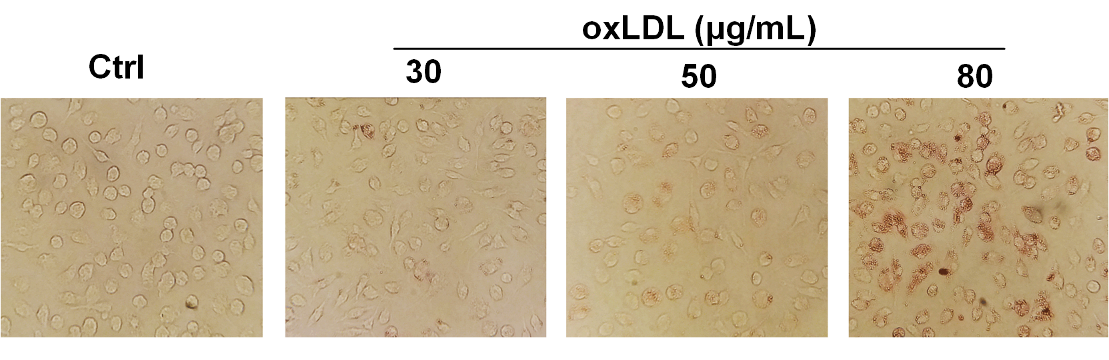
**

**Fig.S1 Determination of optimum concentration of oxLDL.**

Primary mouse peritoneal macrophages were treated with different concentration oxLDL for 24 h. And then washed three times with PBS, fixed with 4% paraformaldehyde for 15 min and then stained with ORO. The intracellular lipid droplets were then detected with optical microscope. Result showed that the higher oxLDL concentration, the more lipid accumulation. Because at the concentration of 80 µg/ml, the lipid accumulation is excessive and the cell state is not good, we chose 50 µg/ml oxLDL in the experiment.

**Supplementary Figure 2**


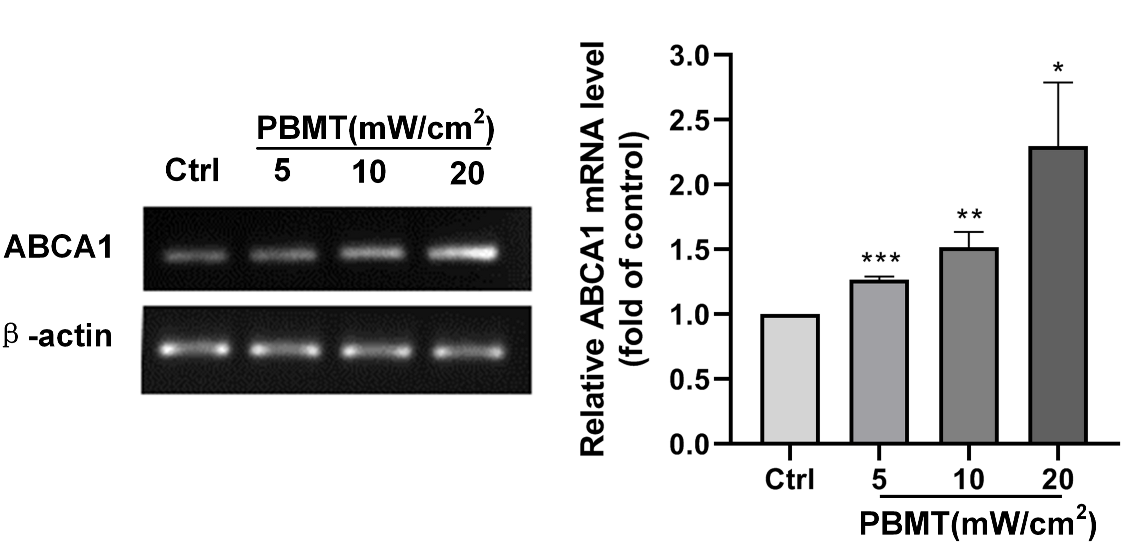


**Fig.S2 Upregulation of ABCA1 by PBMT was dose-dependent.**

Primary mouse peritoneal macrophages were treated with different dose laser and RNA was isolated after 6 h to detect the expression of ABCA1. Upregulation of ABCA1 by PBMT was dose-dependent. 20 mW/cm^2^ was chose as the optimal dose and was used in follow-up experiments.

Data are shown as mean ± SEM, Control vs. PBMT, **P* < 0.05, ***P* < 0.01, ****P* < 0.001, *ns* = not significant, unpaired two tailed Student’s *t* test.

**Supplementary Figure 3**

**
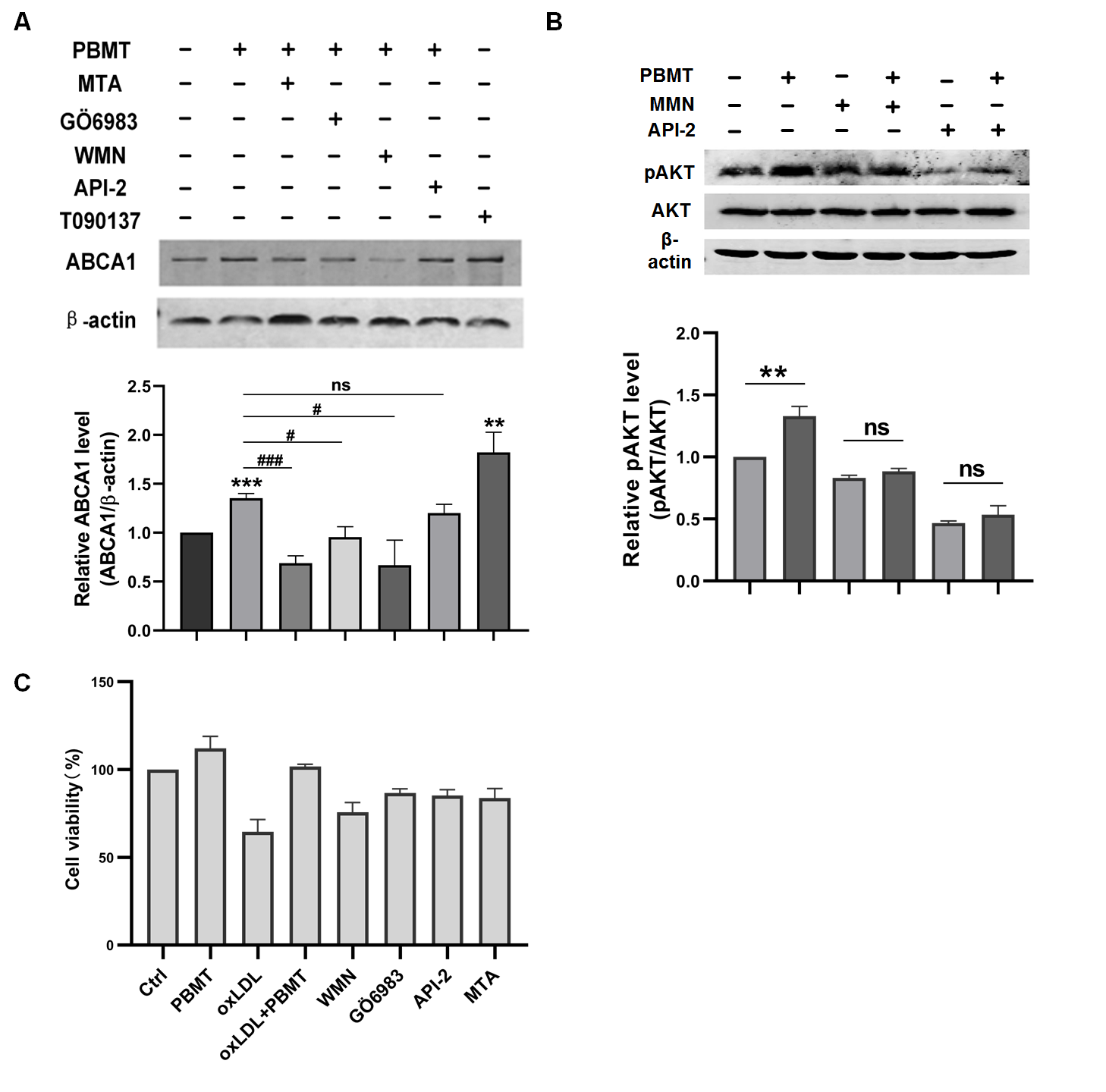
**

**Fig.S3 Upregulation of ABCA1 by PBMT was mediated by SP1, PKCζ， PI3K not AKT.**

Primary mouse peritoneal macrophages were processed differently with PBMT, MTA (SP1 inhibitor), GÖ6983 (PKCζ inhibitor); WMN (PI3K inhibitor); API-2 (AKT inhibitor) and positive treatment T090137.

1. Western blot analysis of ABCA1 expression in primary mouse peritoneal macrophages with different treatments. (*n*=4).
2. Western blot analysis of pAKT level in primary mouse peritoneal macrophages with different treatments. (*n*=4).
3. CCK-8 analysis of cell viability in primary mouse peritoneal macrophages with different treatments. (*n*=6).

Data are shown as mean ± SEM, one-way ANOVA. ^#^*P* < 0.05 ***P* < 0.01, ****P* < 0.001, ^##^*P* < 0.01, ^##^*P* < 0.001, *ns* = not significant.

**Supplementary Table 1**

**Table S1. Mouse primer sequence**

| **Primer** | **Forward sequence** | **Reverse sequence** | **Predicted**  **size** |
| --- | --- | --- | --- |
| **ABCA1** | **GGTTTGGAGATGGTTATACAATAGTTGT** | **CCCGGAAACGCAAGTCC** | **94bp** |
| **ABCG1** | **GCAGATGTGTCAGGACCGAGT** | **TCACCCAGTTCTGCATCCTCTT** | **72 bp** |
| **CD36** | **ATGGGCTGTGATCGGAACTG** | **TTTGCCACGTCATCTGGGTTT** | **233 bp** |
| **LDLR** | **CTCCTGCATTCACGGTAGCC** | **CCCACTGTGACACTTGAACTTG** | **124 bp** |
| **HMGCR** | **CTTGTGGAATGCCTTGTGATTG** | **AGCCGAAGCAGCACATGAT** | **76 bp** |
| **SREBP2** | **GCGTTCTGGAGACCATGGA** | **ACAAAGTTGCTCTGAAAACAAATCA** | **131 bp** |
| **SR-AI** | **TGGAGGAGAGAATCGAAAGCA** | **CTGGACTGACGAAATCAAGGAA** | **140 bp** |
| **SR-BI** | **TGTACTGCCTAACATCTTGGTCC** | **ACTGTGCGGTTCATAAAAGCA** | **126 bp** |
| **β-actin** | **GCACAGCTTCTTTGCAGCTCCTCG** | **TTTGCACATGCCGGAGCCGTTG** | **109 bp** |

**Supplementary Figure 4.**

**Original gel scans**

**
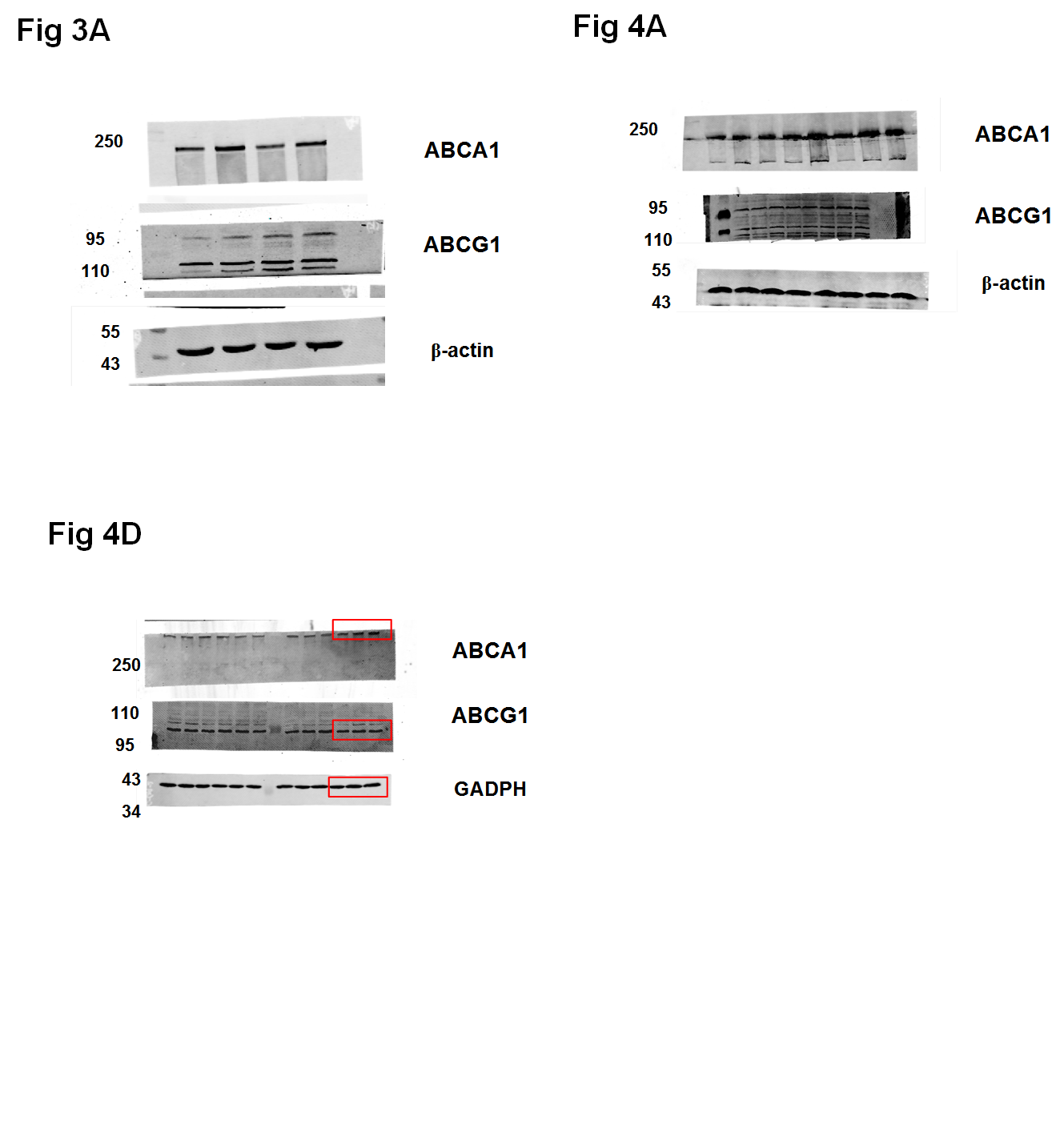
**

**
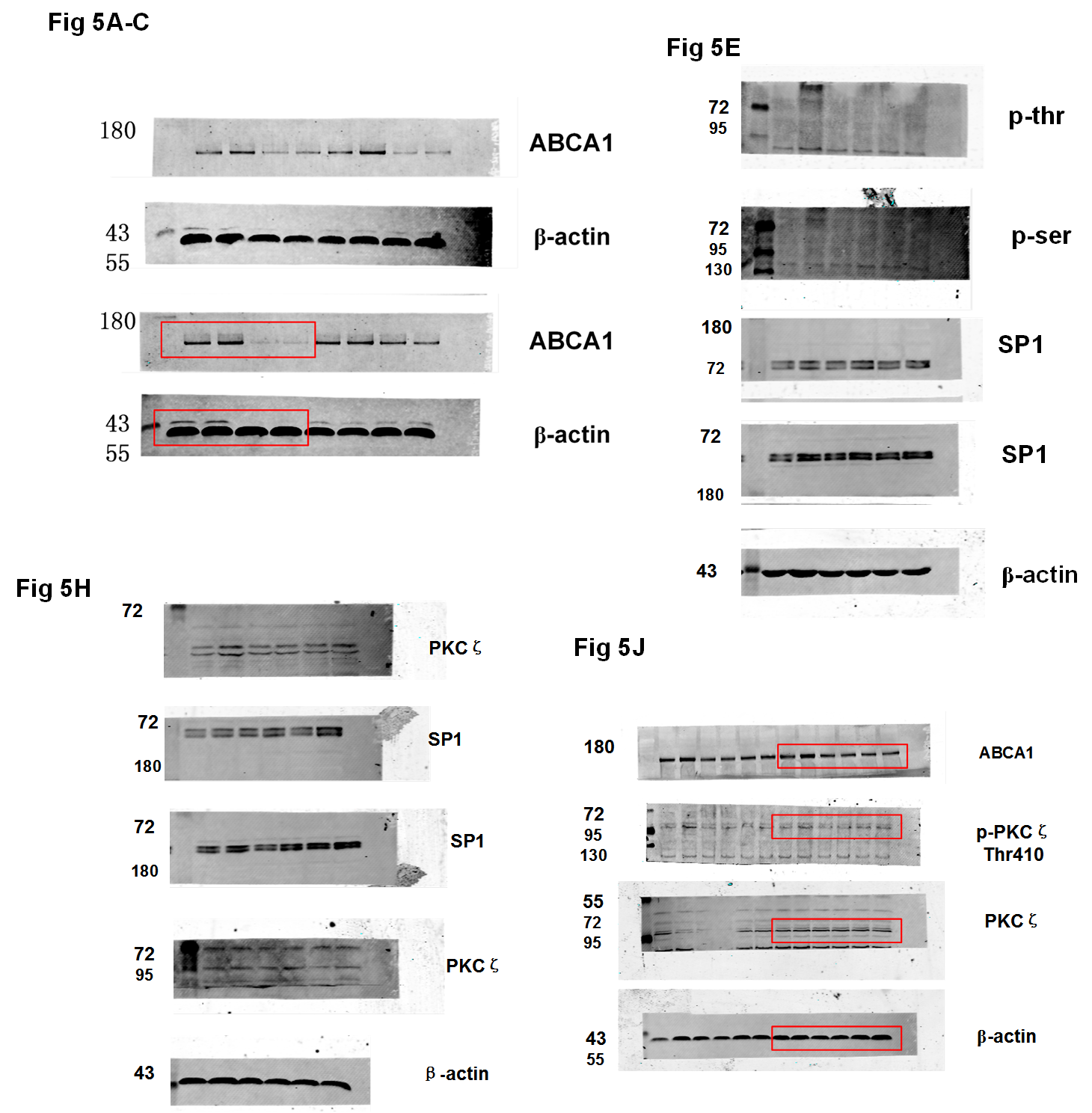
**
